# Respiratory Diseases' Burden in children and adolescents of marginalized population: A retrospective study in slum area of Karachi, Pakistan

**DOI:** 10.3389/fepid.2022.1031666

**Published:** 2023-01-11

**Authors:** Hina Sharif, Shah Sumaya Jan, Sana Sharif, Tooba Seemi, Hira Naeem, Junaid Rehman

**Affiliations:** ^1^ Research & Publication Department, SINA Health & Education Welfare Trust, Karachi, Pakistan; ^2^ Department of Anatomy, Government Medical College, Srinagar, India; ^3^ School of Public Health, University of Saskatchewan, Saskatoon, SK, Canada; ^4^ Public Health Department, SINA Health, Education & Welfare Trust, Karachi, Pakistan

**Keywords:** bronchiolitis, children and adolescents, epidemiology, inadequate ventilation, large family size, pneumonia, seasonal variations, slum areas

## Abstract

**Background:**

Worldwide, the burden of respiratory disease has dramatically increased, endangering public health. To our knowledge, there have been no reported cases of respiratory illness among children and adolescents living in the slums of Karachi, Pakistan. This study aimed to assess the burden of respiratory disease in marginalized slum populations and the factors causing such an increase in disease burden.

**Methods:**

This study was conducted in 35 slums of Karachi, Pakistan, to determine the prevalence of respiratory disease in children and adolescents. Data on pneumonia, bronchitis, bronchiolitis, tuberculosis, and asthma from August 2019 to July 2022 were analyzed and inferences were drawn.

**Results:**

Among the studied diseases, pneumonia was more prevalent among females (39,864, 44.9%), followed by males (19,006, 21.4%). Most of the children (59,988, 67.6%) were aged 1–5 years. In addition, of those diagnosed with pneumonia, 50,348 (56.8%) were from the same age group. Furthermore, bronchiolitis was found among 10,830 (12.2%) children aged 5–9 years. The majority (46,906, 52.9%) of the studied population belonged to the Pathan ethnicity, followed by Sindhi (21,522, 24.2%), and most of them (84,330, 95.1%) were of a lower socioeconomic status.

**Conclusions:**

This study found that pneumonia is the most common respiratory disease followed by bronchiolitis in children and adolescents in a marginalized slum population of Karachi, Pakistan. Both pneumonia and bronchiolitis have seasonal variations in their occurrence.

## Introduction

Pakistan, with a total area of 796,100 km^2^ (307,376 mi^2^), had a population of 224.78 million in 2021, of which 82.83 million lived in urban areas, while 141.96 million lived in rural areas (1). Karachi, one of the most populated cities of Pakistan, is located at the southern tip of the country on the coast of the Arabian Sea; it is sometimes classified as Beta World City as it is Pakistan's major industrial and financial center. The metropolitan hub and its suburbs comprise a mixed population of economic and political migrants and refugees from diverse national, regional, linguistic, and religious backgrounds. Due to the rapid population growth associated with rural-to-urban migration, industrialization, and the economic crisis the country is experiencing, people are bound to live in slums. These slum people are highly vulnerable in terms of low socioeconomic status, high illiteracy, poverty, large family size, poor access to health facilities, and poor living conditions (2, 3). According to recent Karachi surveys, people from six significant ethnic backgrounds reside in slums here, including Urdu-speaking Pathan, Punjabi, Sindhi, Saraiki, and Balochi (4, 5).

Recent research in slum areas has shown that the urban poor perform worse on most health indicators, and the diseases, especially the burden of respiratory disease, are intense (6). Respiratory diseases cause high morbidity and mortality in infants and children worldwide, affecting more than 1 billion people (7–12). Respiratory diseases, such as pneumonia, tuberculosis (TB), acute respiratory infections, chronic obstructive pulmonary disease, asthma, bronchitis, and bronchiolitis, are health threats globally and cause significant harm to children. About 4 million deaths in low- and middle-income countries are mainly due to respiratory diseases (13).

Studies from Pakistan have reported lower respiratory tract infections (LRTI) as the leading cause of death in children and adolescents (14, 15). Among LRTIs, pneumonia is one of the leading causes of death among children aged under 5 years (16). In the United States, pneumonia affects 2.6% of children aged below 17 years (17). In other high-income countries, the yearly frequency of pneumonia is assessed at 33 per 10,000 in children aged below 5 years and 14.5 per 10,000 in children aged 0–16 years (18–20). In addition, acute respiratory illness and diarrhea were among the leading causes of death in infants and children aged under 5 years (21). Bronchiolitis, caused by respiratory syncytial virus (RSV), is the second leading cause of respiratory disease after pneumonia, resulting in hospitalized children aged under 5 years, with a rough estimate of 3.8 million new incidents during 2010 (22). As per the global burden of disease report, Pakistan ranks seventh among the 22 countries, with the highest TB burden globally (23, 24). TB in infants aged below 12 months has a 50% risk of progression to TB if exposed to the TB bacteria. In contrast, children aged 1–2 years have a 20%–30% risk of developing TB. Additionally, children aged 3–5 years have a 5% risk, and those aged 5–10 years have only a 2% risk (25). Bronchitis, an inflammation of the lining of bronchial tubes, is common among children and adolescents and affects up to 40% of newly referred children to specialist care for a persistent productive cough (26, 27). Moreover, data suggest that the growing prevalence of asthma is also a global concern. Approximately 180,000 deaths are related to asthma annually (28).

### Study rationale

To plan for and meet the health needs of a specific population, information about their health status is needed. However, information on health planning is scarce in most low-income countries, partly due to a lack of health infrastructure and knowledge. Data on other health conditions often come from small hospital studies, but most illnesses and deaths occur outside the healthcare system. Estimating the disease burden in a population is one way to generate health information for planning. Although these estimates help make national and international policy decisions, they mask the differences between and within countries and may not be relevant to local health planning (29). The situation is even worse for disadvantaged and marginalized populations, such as the slum population, who have so far received little attention in research and service delivery (30).

Therefore, the aim of the present study was to quantify the common respiratory disease burden prevalent among the slum population of Karachi, Pakistan using secondary data generated from a Hospital management information system (HIMS). The present study evaluates the respiratory disease burden in marginalized slum populations, the factors that cause an increase in this burden, the seasonal exacerbation of the commonest respiratory diseases, and the genders and ethnicities highly affected by this burden. It also evaluates the burden of respiratory disease in marginalized slum populations and the factors causing this increase in disease burden.

### What this study adds

Previously reported data (31) indicate an era when communicable respiratory diseases were favored in many parts of the world. For this reason, various prevention and treatment strategies helped reduce the incidence and prevalence of such infectious diseases in both high- and low-income countries. Today, with newer evolving lifestyle diseases, the burden of non-communicable diseases (NCDs) has increased exponentially in high- and low-income countries. In contrast, infectious diseases have been largely ignored (31). In addition, emerging and remerging infections have contributed to the pool of infectious disease burden. Much of this burden can be prevented or treated with affordable interventions.

Identifying the contemporary epidemiological trends in disease is the key to making informed policy decisions on helpful resource allocations. In addition, the disease trend will reformed policymakers, local governments, international organizations, and the health system about the community's health needs.

## Material and methods

### Study design and site

This is a cross-sectional observational study of a marginalized population in Karachi, Pakistan. We collected and analyzed secondary data from SINA Health, Education & Welfare Trust (SINA) clinics between August 2019 and July 2022. SINA is a privately funded non-profit organization serving slum communities since 1998 through a network of 38 clinic sites, including three mobile vans in slum areas. Together, these clinics serve approximately 1 million people annually. They provide quality primary healthcare to disadvantaged people, especially women, children, and adolescents. As shown in Supplementary Figure S1, SINA clinics are spread throughout Karachi City, Pakistan. Therefore, we regarded it as a representative study setting.

### Urban slum

A slum is defined as a compact area with a population of at least 300, or about 60–70 households, with poorly built and congested tenements in an unhygienic environment, usually with inadequate infrastructure and lacking the proper sanitary and drinking water facilities (32).

### Data management information system

SINA has medical records under the Hospital Management Information System (HIMS), which maintains the data of every patient and disease in detail in the form of an electronic medical record (EMR) system. The retrospective data of the five common respiratory diseases (TB, pneumonia, asthma, bronchitis, and bronchiolitis) for 3 years (August 2019 to July 2022) were acquired on request. On acquisition, the data on the children and adolescent population, whose records were available with the SINA's EMR system, were selected for analysis. A total of 88,693 records of children and adolescents were found for the given study period, among which 28,418 were boys and 60,275 were girls.

### Patient flow at SINA health clinics

Once a patient visits any SINA-affiliated health clinic, they must pass through various processes. All new and follow-up patient records are captured through the electronic management record system at reception after confirming their sociodemographic details. The patient is then referred to the vital sign station to check their vitals, height, and weight and is then assigned a token number to wait their turn to visit the physician or a specialist doctor for consultation. The flow management of patients is shown in Supplementary Figure S2.

### Data acquisition and quality

Patients are allocated a random patient identification number when the data are entered into the database. All information is aggregated to ensure patient anonymity. Therefore, the results are presented as aggregate data. All electronic data are password-protected; only the research team can access that file on request.

### Statistical analysis

The descriptive statistics of the sociodemographic variables were computed as frequencies with percentages. Prevalence was estimated for the presence of chronic respiratory symptoms and illnesses by calculating the frequency and percentages of the occurrence of symptoms and illnesses among children and adolescents. A chi-square test of significance was conducted in order to assess the difference in the frequency of respiratory symptoms and illness among the sexes, seasonal variation, and ethnic groups. The differences were then assessed for their significance by their *p*-values, with *p* = 0.05 or less considered to be significant. All statistical analyses were performed using SPSS Statistics version 27 (IBM Corp., Armonk, NY, United States).

## Results

The sociodemographic details of the study population are described in Supplementary Table S1. In total, 88,693 records of children and adolescents were analyzed for the given study period, among which 28,418 (32.0%) were boys and 60,275 (67.9%) were girls. Among the studied diseases, pneumonia was more prevalent among girls (39,864, 44.9%) compared to boys (19,006, 21.4%). Most of the children (59,988, 67.6%) were aged 1–5 years. In addition, of those diagnosed with pneumonia, 50,348 (56.8%) were from the same age group. Furthermore, bronchiolitis was found among 10,830 (12.2%) children aged 5–9 years. It was also found that most of the studied population (84,330, 95.1%) was from the urban region, among which 56,406 (63.6%) were diagnosed with pneumonia, followed by bronchitis 24,178 (27.2%). The majority of the study population (46,906, 52.9%) belonged to the Pathan ethnicity, followed by Sindhi (21,522, 24.2%); most of them (84,330, 95.1%) were of a lower socioeconomic status. The majority (72,859, 82.1%) had an income under 13,000 Pakistani rupees per month.

Seasonal variation in the prevalence of disease among the studied population (per 10,000 population) is described in Supplementary Table S2. It has been found that pneumonia was most prevalent in the winters of 2019 (448 per 10,000 population) and 2021 (4.91 per 10,000 population), while bronchiolitis was more prevalent in the summers of 2020 (2.81 per 10,000 population), 2021 (3.13 per 10,000 population), and 2022 (5.74 per 10,000 population). Moreover, bronchitis was more prevalent in the winter of 2019 (2.76 per 10,000 population) and the monsoon season of 2021 (5.74 per 10,000 population).

The association between age, region, ethnicity, and seasons with respiratory diseases among the studied population is described in Supplementary Table S3. It has been found that all variable ages, regional differences, ethnic variations, and seasonal patterns influence the development of the disease within the studied population, and the association was statistically significant with a *p*-value <0.05 at a 95% confidence interval.

## Discussion

There are few studies on respiratory epidemiology from Pakistan, and there is no reliable database on the distribution of respiratory disorders among a slum population. This is the first study that has investigated the prevalence of respiratory symptoms in the context of pneumonia, bronchiolitis, bronchitis, TB, and asthma in more than 80,000 people living in slums, mostly children and adolescents, and has shown a high prevalence of these respiratory morbidities in the slum community. Sex, age under 5 years, regional disparity, ethnicity, and seasonal variations were significant risk factors associated with mainly pneumonia and bronchitis in the slum dwellers. It should be mentioned here that Pathans and Sindhis people were mostly affected, and the winter season was more favorable for the development of pneumonia while summers were more favorable for bronchiolitis, and they emerged as significant risk factors associated with symptoms of these respiratory diseases.

According to reports, Bangladesh, India, Indonesia, and Nepal account for 40% of all global deaths due to respiratory disease (33). Acute respiratory diseases are the third most frequent cause of death for an individual worldwide, whether in high- or low-income countries (34). The range of reporting research studies for the prevalence of respiratory disease is 20%–30% (35–37). The present study, like the other investigations, estimated that the prevalence of respiratory disease among children aged under 5 years was 68.9%, among which pneumonia contributed to 56.8% of cases. Research from the Indian state of Karnataka found that the frequency of respiratory illness in children aged under 5 years was approximately 10% (38). The prevalence of respiratory illness varies depending on variations in the social, cultural, and environmental elements present in the various geographic regions. According to other studies, the prevalence is higher (39, 40).

There was no association of exposure to unhealthy fuels with asthma, TB, or bronchiolitis in the slum population. This study reported a low prevalence of asthma (0.6%), TB (0.1%), and bronchiolitis (2.7%), with a higher prevalence of bronchitis (28.7%) in the slum population, which is similar to previous reports from the Indian subcontinent (41, 42).

Annually, there are over 120 million cases of pneumonia worldwide, and 1.7 million people die from the disease (43). Many of the deaths might be avoided with easy steps and could be treated with affordable treatment and technology. In the present study, the prevalence of pneumonia is highly correlated with lower age, ethnicity, socioeconomic status, and season. One risk factor for respiratory diseases is a lower income, which was confirmed by a study conducted in the Indian State of West Bengal's Hooghly district and yielded similar results (44).

In our study, we found that slum dwellers from the Punjabi, Balochi, and Saraiki ethnicities are less likely to have a respiratory disease burden than Pathans and Sindhis. This may be due to many factors, such as ethnicity, that contribute to the illness load. Our findings confirm the ethnic relationship to disease burden, because the Pathans and Sindhis in Karachi are primarily laborers (45) who have a lower-standard lifestyle, frequently use tobacco and *naswar* at home (46), and have overcrowded homes. Children and adolescents, especially those aged under 5 years, who live in homes with inadequate ventilation are more likely to develop respiratory infections than those who live in homes with adequate ventilation, which is another major factor contributing to the rise in respiratory diseases among slum dwellers (47).

Although often unpredictable, seasonal change may have an impact on the frequency and geographic distribution of the disease pattern. In our study, pneumonia was most prevalent in the winters of 2019 (448 per 10,000 cases) and 2021 (4.91 per 10,000 cases), while bronchiolitis was more prevalent in the summers of 2020 (2.81 per 10,000 cases), 2021 (3.13 per 10,000 cases), and 2022 (5.74 per 10,000 cases). In addition, bronchitis was also prevalent in the winter of 2019 (2.76 per 10,000 cases) and the monsoon season of 2021 (5.74 per 10,000 cases). Although seasonal variations do exist with other diseases, such as influenza, as reported in studies (48), we could not find any local study from Pakistan that could establish this relationship.

### Strengths of the study

First, a vulnerable group of children and adolescents from Karachi, Pakistan's heavily populated urban slum areas, were included in this community-based multi-site study. This is the first study on children and adolescents in age- and ethnicity-specific marginalized population. Large study sample size is one of the biggest strengths of the study which has been taken over the period of three years. The data obtained from SINA' s HMIS was validated and without any missing value or error. The authors' private, nonprofit Institute epitomizes the positive relationship and liaising between the public and private sectors.

### Limitations of the study

The cross-sectional nature of the study and the analysis of the secondary data are the biggest limitations of the study. Other variables, such as the literacy of the mothers, housing conditions, vaccination status, smoking status, environmental conditions, and malnutrition among children, were not analyzed during the study, which was quite relevant to the objectives.

## Conclusion

Pneumonia is a common childhood illness in low-income nations such as Pakistan, with risk factors including age under 5 years, ethnicity, regional differences, seasonal variation, and lower socioeconomic status, which is a situation typically seen in urban slums. Effective community-based interventions, such as health education and raising awareness among slum dwellers, may significantly reduce the risk.

## Data Availability

The raw data supporting the conclusions of this article will be made available by the authors, without undue reservation.
