# Supplementary material for: Respiratory Diseases' Burden in children and adolescents of marginalized population: A retrospective study in slum area of Karachi, Pakistan
Source: Front Epidemiol. 2023 Jan 11;2:1031666. doi: 10.3389/fepid.2022.1031666 (PMC10911041; doi:10.3389/fepid.2022.1031666)

Supplementary Material

**Supplementary Figures and Tables:**

**Table 1:** Demographics of the study population.

|  | **Diseases** | **Pneumonia** | **Bronchiolitis** | **Bronchitis** | **Tuberculosis** | | **Asthma** |
| --- | --- | --- | --- | --- | --- | --- | --- |
| **Variables** | **Frequency N (%)** | **n (%)** | | | | | |
| **Gender** | | | | | | | |
| - Male | 28387(32.0) | 19006 (21.4) | 1317 (1.4) | 7182 (8.1) | | 78 (0.08) | 804 (0.9) |
| - Female | 60250(67.9) | 39864 (44.9) | 1163 (1.3) | 18316(20.6) | | 203 (0.2) | 704 (0.7) |
| **Age** | | | | | | | |
| - >1-<12 months (infant) | 1156 (1.3) | 1106 (1.2) | 50 (0.05) | 0 (0) | | 0 (0) | 0 (0) |
| - >1y-<5y | 59988(67.6) | 50348 (56.8) | 9550 (10.7) | 0 (0) | | 55 (0.06) | 35 (0.03) |
| - >5y-<9y | 16840(18.9) | 5720 (6.4) | 10830 (12.2) | 0 (0) | | 172 (0.1) | 118 (0.1) |
| - >10y-<14y | 5983 (6.7) | 1281 (1.4) | 3487 (3.9) | 787 (0.8) | | 37 (0.04) | 391 (0.4) |
| - >15y-<19y | 4670 (5.2) | 415 (0.4) | 1581 (1.7) | 1693 (1.9) | | 17 (0.01) | 964 (1.0) |
| **Regions** | | | | | | | |
| - Urban | 84330(95.1) | 56406 (63.6) | 2180 (2.4) | 24178(27.2) | | 265 (0.2) | 1301 (1.4) |
| - Rural | 4307 (4.8) | 2464 (2.7) | 300 (0.3) | 1320 (1.4) | | 16 (0.01) | 207 (0.2) |
| **Ethnicity** | | | | | | | |
| - Pathan | 46906(52.9) | 34000 (38.3) | 980 (1.1) | 11100(12.5) | | 122 (0.1) | 704 (0.7) |
| - Sindhi | 21522(24.2) | 12000 (13.5) | 850 (0.9) | 8016 (9.0) | | 96 (0.1) | 560 (0.6) |
| - Punjabi | 3943 (4.4) | 3000 (3.3) | 130 (0.1) | 778 (0.8) | | 10 (0.01) | 25 (0.02) |
| - Balochi | 12084(13.6) | 7000 (7.8) | 350 (0.3) | 4500 (5.0) | | 40 (0.04) | 194 (0.2) |
| - Saraiki | 2865 (3.2) | 2006 (2.2) | 50 (0.05) | 782 (0.8) | | 7 (0.0) | 20 (0.02) |
| - Other | 1317 (1.4) | 864 (0.9) | 120 (0.1) | 322 (0.3) | | 6 (0.0) | 5 (0.0) |
| **Socioeconomic Status** | | | | | | | |
| - Upper | 0 (0) | 0 (0) | 0 (0) | 0 (0) | | 0 (0) | 0 (0) |
| - Upper middle | 0 (0) | 0 (0) | 0 (0) | 0 (0) | | 0 (0) | 0 (0) |
| -Lower middle | 4307 (4.8) | 2464 (2.7) | 300 (0.3) | 1320 (1.4) | | 16 (0.01) | 207 (0.2) |
| - lower | 84330(95.1) | 56406 (63.6) | 2180 (2.4) | 24178(27.2) | | 265 (0.2) | 1301 (1.4) |
| **Household Income (In Pakistani Rupee)** | | | | | | | |
| -None | 11471 (12.9) | 6906 (7.7) | 1000 (1.1) | 3178 (3.5) | | 85 (0.09) | 302 (0.3) |
| -Less than 13000 | 72859(82.1) | 49500 (55.8) | 1180 (1.3) | 21000(23.6) | | 180 (0.2) | 999 (1.1) |
| -13000-39000 | 4307 (4.8) | 2464 (2.7) | 300 (0.3) | 1320 (1.4) | | 16 (0.01) | 207 (0.2) |
| -40000-64000 | 0 (0) | 0 (0) | 0 (0) | 0 (0) | | 0 (0) | 0 (0) |
| - more than 65000 | 0 (0) | 0 (0) | 0 (0) | 0 (0) | | 0 (0) | 0 (0) |

**Table 2:** Seasonal Variation in the disease prevalence among the studied population (per 10,000 population)

| **Disease** | **Year** | **Prevalence Rate per 10,000 Population** | | | |
| --- | --- | --- | --- | --- | --- |
|  |  | **Rain** | **Fall** | **Winter** | **Summer** |
|  |  | **(July-Sept)** | **(Oct-Nov)** | **(Dec-Feb)** | **(March-June)** |
| **Pneumonia** | 2019 | 176 | 207 | 448 |  |
|  | 2020 | 8 | 40 | 95 | 243 |
|  | 2021 | 211 | 279 | 491 | 49 |
|  | 2022 |  |  |  | 381 |
|  |  |  |  |  |  |
| **Bronchiolitis** | 2019 | 168 | 127 | 159 |  |
|  | 2020 | 141 | 19 | 54 | 281 |
|  | 2021 | 249 | 161 | 234 | 313 |
|  | 2022 |  |  |  | 574 |
|  |  |  |  |  |  |
| **Bronchitis** | 2019 | 72 | 26 | 276 |  |
|  | 2020 | 90 | 10 | 24 | 18 |
|  | 2021 | 574 | 7 | 9 | 29 |
|  | 2022 |  |  |  | 7 |
|  |  |  |  |  |  |
| **Tuberculosis** | 2019 | 0 | 1 | 0 |  |
|  | 2020 | 0 | 0 | 0 | 2 |
|  | 2021 | 1 | 1 | 1 | 0 |
|  | 2022 |  |  |  | 6 |
|  |  |  |  |  |  |
| **Asthma** | 2019 | 8 | 7 | 10 |  |
|  | 2020 | 2 | 3 | 4 | 5 |
|  | 2021 | 6 | 6 | 5 | 6 |
|  | 2022 |  |  |  | 6 |

The gray-shaded areas are out of sample range, i.e., sample were taken from Fall 2019 to Summer 2022

**Table 3:** Association of Age, Region, Ethnicity, and Seasons with Respiratory diseases among the studied population

| **Disease** | **Pneumonia** | | | **Bronchitis** | | | **Bronchiolitis** | | | **Tuberculosis** | | | **Asthma** | | |
| --- | --- | --- | --- | --- | --- | --- | --- | --- | --- | --- | --- | --- | --- | --- | --- |
| **Variables** | **n** | **Male/ Female** | *χ*^2,^  p value | **n** | **Male/ Female** | *χ*^2,^  p value | **n** | **Male/ Female** | *χ*^2,^  p value | **n** | **Male/ Female** | *χ*^2,^  p value | **n** | **Male/ Female** | *χ*^2,^  p value |
| **Age** | | | | | | | | | | | | | | | |
| - >1-<12 months (infant) | 1,106 | 318 / 788 | *χ*^2^ = 44*, <0.05** | 0 | 0 / 0 | χ2 = 193.58,  <0.05* | 50 | 19 / 31 | χ2 = 20,  <0.05* | 0 | 0 / 0 | χ2 = 51,  <0.05* | 0 | 0 / 0 | χ2 =169,  < 0.05* |
| - >1y-<5y | 50,348 | 16508 / 33840 |  | 0 | 0 / 0 |  | 9,550 | 2713 / 6837 |  | 55 | 11.0/44 |  | 35 | 20 / 15 |  |
| - >5y-<9y | 5,720 | 1687 / 4033 |  | 0 | 0 / 0 |  | 10,830 | 3113 / 7717 |  | 172 | 46 / 126 |  | 118 | 20 / 98 |  |
| - >10y-<14y | 1,281 | 356 / 925 |  | 787 | 257 / 530 |  | 3,487 | 962 / 2525 |  | 37 | 4.0/33 |  | 391 | 304 / 87 |  |
| - >15y-<19y | 415 | 137 / 278 |  | 1,693 | 1060 / 633 |  | 1,581 | 375 / 1206 |  | 0 | 17/17 |  | 964 | 460 / 504 |  |
| **Regions** | | | | | | | | | | | | | | | |
| - Urban | 56,406 | 18406 / 38000 | *χ*^2^ = 74*, <0.05** | 2,180 | 1017 /1163 | χ2 = 301,  <0.05* | 24,178 | 5982 / 18196 | χ2 = 270,  <0.05* | 265 | 70 / 195 | χ2 = 4,  <0.05* | 1,301 | 684 / 617 | χ2 = 205,  >0.05* |
| - Rural | 2,464 | 600 / 1864 |  | 300 | 300 / 0 |  | 1,320 | 1200 / 120 |  | 16 | 8.0/8 |  | 207 | 120 / 87 |  |
| **Ethnicity** | | | | | | | | | | | | | | | |
| - Pathan | 34,000 | 13000 / 21000 | *χ*^2^ = 228*, <0.05** | 980 | 480 / 500 | χ2 = 155,  <0.05* | 11,100 | 2800 / 8300 | χ2 = 990,  <0.05* | 122 | 122 / 100 | χ2 = 51,  <0.05* | 704 | 304 / 400 | χ2 = 69,  < 0.05* |
| - Sindhi | 12,000 | 3000 / 12000 |  | 850 | 450 / 400 |  | 8,016 | 2000 / 6016 |  | 96 | 26 / 70 |  | 560 | 360 / 200 |  |
| - Punjabi | 3,000 | 1000 / 2000 |  | 130 | 100 / 30 |  | 778 | 578 / 200 |  | 10 | 10.0/0 |  | 25 | 10.0/15 |  |
| - Balochi | 7,000 | 2000 / 5000 |  | 350 | 250 / 100 |  | 4,500 | 1500 / 3000 |  | 40 | 10.0/30 |  | 194 | 120 / 74 |  |
| - Saraiki | 2,006 | 06.0 / 2000 |  | 50 | 17 / 33 |  | 782 | 182 / 600 |  | 7 | 4.0/3 |  | 20 | 10.0/10 |  |
| - Other | 864 | 0 / 864 |  | 120 | 20 / 100 |  | 322 | 122 / 200 |  | 6 | 6.0/0 |  | 5 | 0 / 5 |  |
| **Season** | | | | | | | | | | | | | | | |
| - Rain | 8,800 | 3041 / 5759 | *χ*^2^ = 99,  *<0.05** | 558 | 230 / 328 | χ2 = 452,  <0.05* | 16,598 | 542 / 16056 | χ2 = 169  , <0.05* | 25 | 25.0/0 | χ2 = 117,  <0.05* | 334 | 153 / 181 | χ2 = 10,  < 0.05* |
| - Autumn | 11,708 | 3522 / 8186 |  | 307 | 286 / 21 |  | 948 | 721 / 227 |  | 24 | 18/6 |  | 371 | 201 / 170 |  |
| - Winter | 22,984 | 7132 / 15852 |  | 447 | 353 / 94 |  | 6,722 | 5711 / 1011 |  | 42 | 13/29 |  | 410 | 233 / 177 |  |
| - Summer | 15,378 | 5311 / 10067 |  | 1168 | 448 / 720 |  | 1,230 | 208 / 1022 |  | 190 | 22 / 168 |  | 393 | 217 / 176 |  |

**Figure-1:** SINA Clinics Map covering almost whole Karachi Slums Area (image taken from the SINA profile).


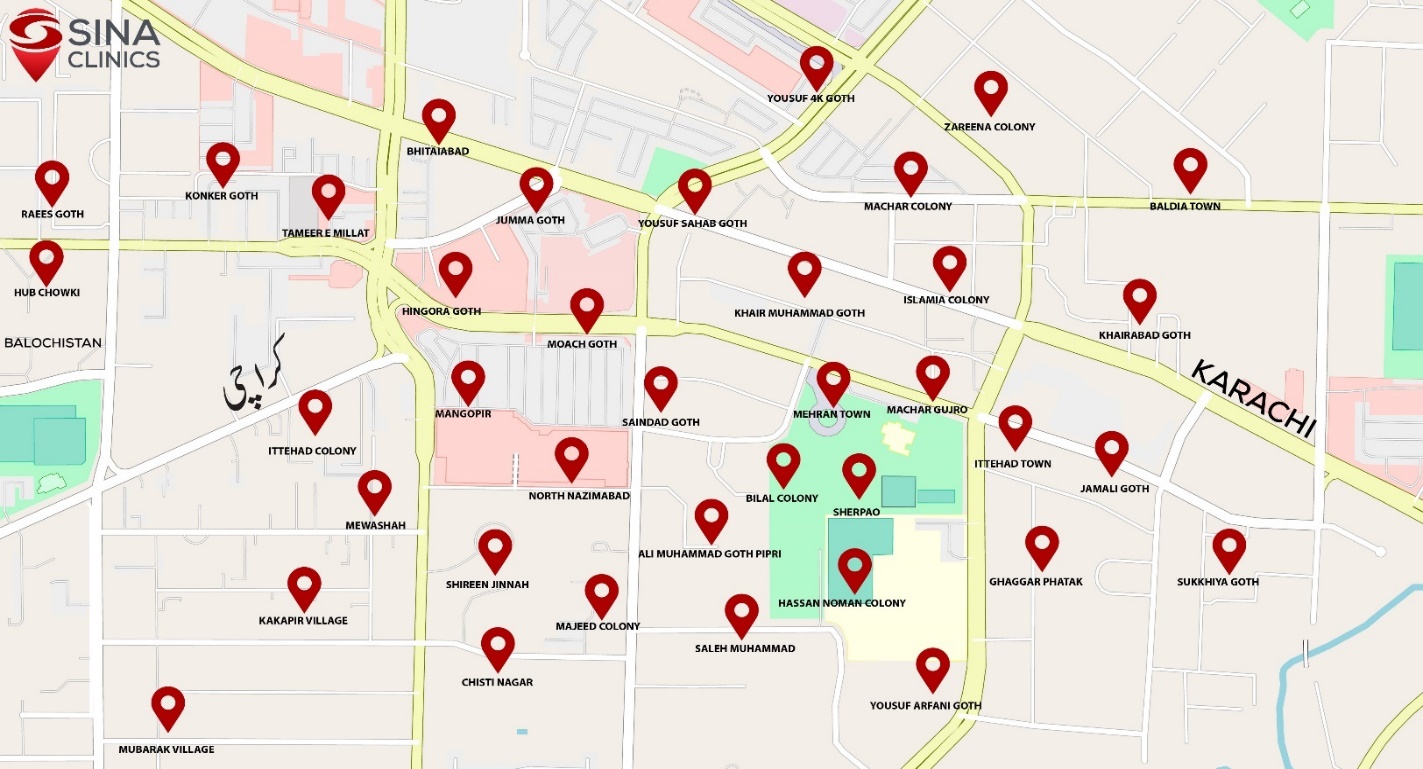


**Figure-2: Patient flow management at SINA clinics.**


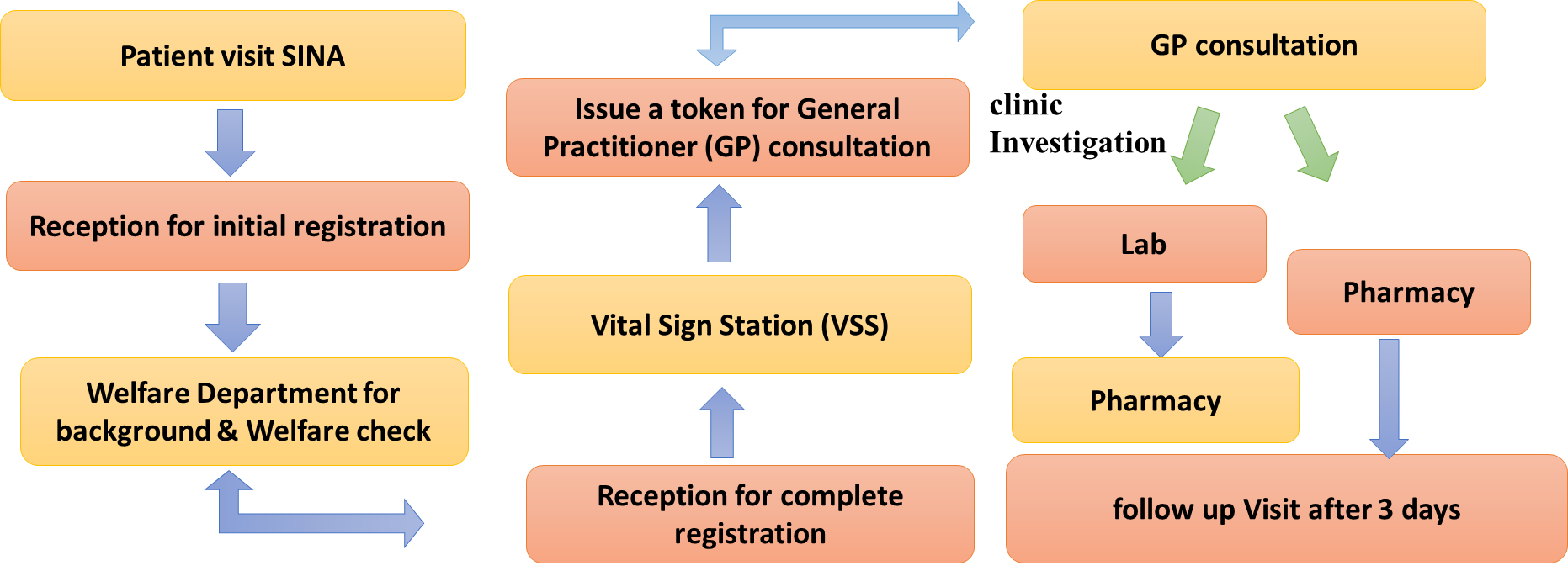


**Figure-3:** Seasons in Karachi, Pakistan

**.**
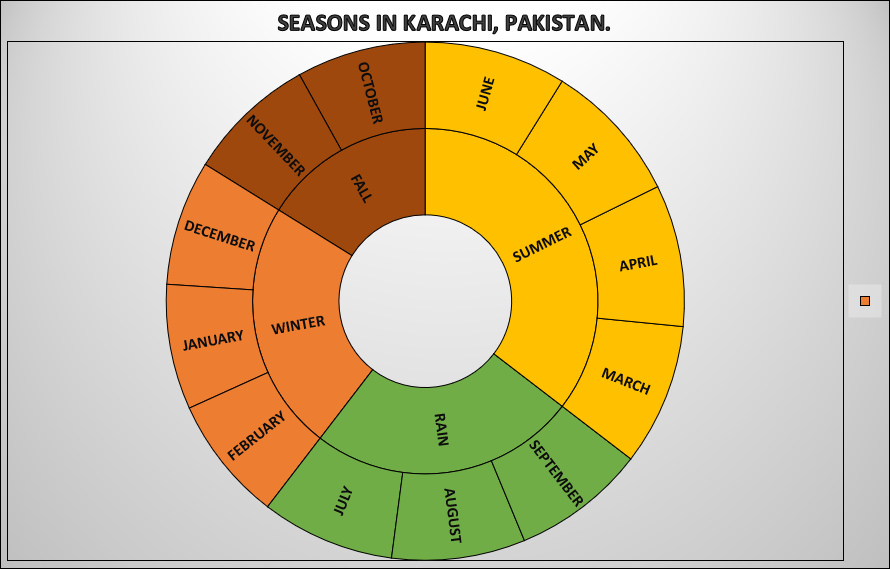

Supplement: Supplementary file 1 [file Table1.docx]
